# Supplementary material for: Twist-angle dependence of moiré excitons in WS2/MoSe2 heterobilayers
Source: Nat Commun. 2020 Nov 18;11:5888. doi: 10.1038/s41467-020-19466-6 (PMC7675978; doi:10.1038/s41467-020-19466-6)
Supplement: Supplementary file 1 — Supplementary Information [file 41467_2020_19466_MOESM1_ESM.pdf]

Twist-angle dependence of moiré excitons in WS_2/$MoSe_2$ heterobilayers

Long Zhang[1],Zhe Zhang[1,2], Fengcheng Wu[3],[*] Danqing Wang[1], Rahul Gogna[4], Shaocong Hou[5], Kenji Watanabe[6], Takashi Taniguchi[6], Krishnamurthy Kulkarni[5], Thomas Kuo[1], Stephen R. Forrest[1,5], and Hui Deng[1,4†]

[1] *Physics Department, University of Michigan, 450 Church Street, Ann Arbor, MI 48109-2122, USA*

[2] *State Key Laboratory of Surface Physics, Department of Physics, Fudan University, Shanghai 200433,China*

[3] *Condensed Matter Theory Center and Joint Quantum Institute, Department of Physics, University of Maryland, College Park, Maryland 20742, USA*

[4] *Applied Physics Program, University of Michigan, 450 Church Street, Ann Arbor, MI 48109-1040, USA*

[5] *Department of Electrical Engineering and Computer Science, University of Michigan, 450 Church Street, Ann Arbor, MI 48109-1040, USA and*

[6] *National Institute for Materials Science, Tsukuba, Japan*

[*] wufcheng@umd.edu

[†] dengh@umich.edu

Supplementary Note 1. Coupled oscillator model on hybrid excitons

To extract the coupling strength J and detuning δ of intralayer and interlayer excitons, we use the coupled oscillator model to describe the exciton hybridization, and write the Hamiltonian as:

$$H = \begin{bmatrix} E_{\mathrm{IX}} & J \\ J & E_{\mathrm{X}} \end{bmatrix}$$

where E_{IX} and E_{X} represent the energies of uncoupled interlayer exciton and intralayer exciton, and J is the tunneling strength of conduction bands electrons. By diagonalizing the matrix, the eigen energies can be extracted, and difference of the two eigen states is $\Delta E = \sqrt{4J^2 + \delta^2}$, where $\delta = E_{\mathrm{IX}} - E_{\mathrm{X}}$. Since the oscillator strength of uncoupled interlayer exciton state is negligibly small compared with the intralayer exciton state, we set it as 0 in the calculation. The ratio of the oscillator strength between the hybrid excitons is $\frac{f_{\mathrm{LHX}}}{f_{\mathrm{UHX}}} = \frac{\sqrt{\delta^2+4J^2}+\delta}{\sqrt{\delta^2+4J^2}-\delta}$.

Supplementary Note 2. Theory for hybrid moiré excitons

We present a microscopic theory to account for our experimentally observed phenomena of hybrid moiré excitons. For definiteness, here we focus on R-stacking bilayers with a small twist angle θ near 0°, and the hybrid MoA states at K valley. The corresponding intralayer A exciton state of $\mathrm{MoSe_2}$ can be represented as

$$|\mathrm{X}\rangle = \frac{1}{\sqrt{\mathcal{A}}} \sum_{\mathbf{k}} \psi_{\mathbf{k}} a^{\dagger}_{c,M,(\mathbf{K}_M+\mathbf{k})} a_{v,M,(\mathbf{K}_M+\mathbf{k})} |G\rangle, \tag{1}$$

where $|G\rangle$ represents the ground state of the system with fully filled valence bands, and $a^{\dagger}_{c,M,(\mathbf{K}_M+\mathbf{k})} a_{v,M,(\mathbf{K}_M+\mathbf{k})}$ creates a particle-hole excitation at valley K in $\mathrm{MoSe_2}$ layer, and $\psi_{\mathbf{k}}$ is the relative-motion wave function. In Eq. (1), $\mathcal{A}$ is the system area, $\mathbf{k}$ is the relative momentum between electron and holes, and $\mathbf{K}_M$ is the momentum of the K point in the Brillouin zone of monolayer $\mathrm{MoSe_2}$. The exciton state $|\mathrm{X}\rangle$ in Eq. (1) has a zero center-of-mass momentum, and therefore, can couple directly to the light and lead to optical absorption.

For interlayer excitons, we consider states with a generic finite center-of-mass momentum $\mathbf{Q}$[9]

$$|\mathbf{Q}\rangle_{\mathrm{IX}} = \frac{1}{\sqrt{\mathcal{A}}} \sum_{\mathbf{k}} \phi_{\mathbf{k}} a^{\dagger}_{c,W,(\mathbf{K}_W+\mathbf{k}+\frac{m_{\mathrm{e,IX}}}{M_{\mathrm{IX}}}\mathbf{Q})} a_{v,M,(\mathbf{K}_M+\mathbf{k}-\frac{m_{\mathrm{h,IX}}}{M_{\mathrm{IX}}}\mathbf{Q})} |G\rangle, \tag{2}$$

where $a_{v,M,(\mathbf{K}_M+\mathbf{p})}$ and $a^{\dagger}_{c,W,(\mathbf{K}_W+\mathbf{p}')}$ respectively create a hole in $\mathrm{MoSe_2}$ valence band and an electron in $\mathrm{WS_2}$ conduction band. $\phi_{\mathbf{k}}$ is the corresponding wave function, and $\mathbf{k}$ is the relative

momentum. $m_{\rm e,IX}$ and $m_{\rm h,IX}$ are respectively electron and hole mass in the interlayer exciton, and $M_{\rm IX} = m_{\rm e,IX} + m_{\rm h,IX}$ is the exciton total mass. The interlayer exciton state $|\mathbf{Q}\rangle_{\rm IX}$ has an energy $\hbar\omega_0 + \hbar^2\mathbf{Q}^2/(2M_{\rm IX})$, which includes an energy constant $\hbar\omega_0$ and a kinetic energy of the center-of-mass motion. In Eq. (2), $\mathbf{K}_W$ is the momentum of the K point in the Brillouin zone of monolayer WS_2, and differs from $\mathbf{K}_M$ due to lattice constant mismatch and misalignment.

The hybridization between intralayer and interlayer excitons is due to interlayer conduction-band tunneling [1] in the moiré pattern, which is given in K-valley by:

$$H_{\rm T} = w\sum_{\mathbf{k}}\sum_{n=1,2,3} a^{\dagger}_{c,W,(\mathbf{K}_W+\mathbf{k}+\mathbf{q}_n)} a_{c,M,(\mathbf{K}_M+\mathbf{k})} + \text{H.c.}, \tag{3}$$

where w is a tunneling parameter. In Eq. (3), $\mathbf{q}_1$, $\mathbf{q}_2$ and $\mathbf{q}_3$ are momenta that compensates the momentum shift between $\mathbf{K}_W$ and $\mathbf{K}_M$ and are connected by the moiré reciprocal lattice vectors. $\mathbf{q}_1$ is equal to $\mathbf{K}_M - \mathbf{K}_W$, while $\mathbf{q}_2$ and $\mathbf{q}_3$ are respectively related to $\mathbf{q}_1$ by $2\pi/3$ and $4\pi/3$ rotations. $|\mathbf{q}_1|$ is given by $4\pi/(3a_M)$, and a_M is the moiré period approximated by $a_0/\sqrt{\theta^2+\varepsilon^2}$, where a_0 is the monolayer lattice constant, and ε is the lattice constant mismatch $|a_0 - a_0'|/a_0$ between the two layers[2]. In Eqs. (1), (2) and (3), the spin label is not shown explicitly, and we consider spin up states.

This interlayer tunneling Hamiltonian $H_{\rm T}$ hybridizes an intralayer exciton $|{\rm X}\rangle$ with an interlayer exciton $|{\rm IX}\rangle$, which shares the same angular momentum as $|{\rm X}\rangle$ and can be written as [3]:

$$|{\rm IX}\rangle = \frac{1}{\sqrt{3}}\Big(|\mathbf{q}_1\rangle_{\rm IX} + |\mathbf{q}_2\rangle_{\rm IX} + |\mathbf{q}_3\rangle_{\rm IX}\Big). \tag{4}$$

The energy difference δ between $|{\rm IX}\rangle$ and $|{\rm X}\rangle$ is

$$\begin{aligned}\delta &= E_{\rm IX} - E_{\rm X} \\ &= \delta_0 + \frac{\hbar^2\mathbf{q}_1^2}{2M_{\rm IX}} \\ &= \tilde{\delta}_0 + \Big(\frac{4\pi}{3a_0}\Big)^2\frac{\hbar^2\theta^2}{2M_{\rm IX}},\end{aligned} \tag{5}$$

where the energy $E_{\rm X}$ of intralayer exciton $|{\rm X}\rangle$ is assumed to be independent of the twist angle θ, while the energy $E_{\rm IX}$ of interlayer exciton $|{\rm IX}\rangle$ increases with increasing θ due to its kinetic energy. Equation (5) provides a quantitative description of the experimentally observed θ dependence of the detuning δ when θ is small.

The coupling J between $|{\rm IX}\rangle$ and $|{\rm X}\rangle$ due to the interlayer tunneling is

$$J = \langle{\rm IX}|H_{\rm T}|{\rm X}\rangle = \frac{\sqrt{3}w}{\mathcal{A}}\sum_{\mathbf{k}}\phi^*_{\mathbf{k}+\frac{m_{\rm h,IX}}{M_{\rm IX}}\mathbf{q}_1}\psi_{\mathbf{k}}, \tag{6}$$

which has been discussed in the main text (See Equation (2) of the main text).

We make two remarks about the theory. (1) While R stacking configuration and the MoA hybrid exctions are assumed in the above analysis, Equations (5) and (6) apply equally well to H stacking configuration and other hybrid excitons, but the exact parameter values can be different for different cases. (2) Moiré pattern can in principle lead to additional bright exciton states besides those that are studied above. Here we only consider hybrid exciton states made of $|X\rangle$ and $|IX\rangle$, because this type of hybrid states have the largest oscillator strengths. In summary, the theory presented here lays a microscopic foundation for the phenomenological coupled oscillator model, provides a microscopic explanation for the experimentally observed θ dependence of J and δ, and allows us to estimate the interlayer exciton total mass M_{IX} and the interlayer tunneling w from the optical spectra.

Supplementary Note 3. Measuring the twist angle of the heterobilayers

The twist angle between the WS_2 and $MoSe_2$ layers is determined by polarization-dependent second-harmonic-generation measurements. Samples are excited by 150 fs pulses (1 MHz repetition) at a wavelength of 860 nm and power of 400 μW with a spot size of 2 μm focused by an objective lens with a numerical aperture of 0.6. More details of the measurements are described in [4]. The SHG patterns from the monolayers, and heterobilayers are shown in Fig. 1. We obtain the crystal orientations by fitting the angular dependence of the intensity pattern with the function: $I_\alpha = cos(3(\alpha + \phi))^2$, where α is the angle between the laser polarization and the armchair edge of the crystal, and the fitting parameter ϕ represents the crystal orientation. The fitting uncertainty corresponds to the 95% confidence interval of the fits. We typically get $\delta\phi_{\text{fit}} = 0.14^\circ$ for both WS_2 and $MoSe_2$. The uncertainty of ϕ should also include the typical variation in ϕ across the sample, measured to be about $\delta\phi_{\text{inh}} = 0.1^\circ$ (shown in Fig. 2c and d), leading to $\delta\phi = \sqrt{\delta\phi_{\text{fit}}^2 + \delta\phi_{\text{inh}}^2}$ with typical values about 0.17°. We obtain the twist angle θ between the two layers by: $\theta = |\phi_1 - \phi_2|$ or $\theta = 60^\circ - |\phi_1 - \phi_2|$, where ϕ_1 and ϕ_2 are from the two monolayers respectively, and the uncertainty of the twist angle can be extracted by $\delta\theta = \sqrt{\delta\phi_1^2 + \delta\phi_2^2}$ with typical values about 0.25°. The uncertainty $\pm\delta\theta$ is plotted in Fig. 2c in the main text for visual comparison with the fit of detuning δ vs. θ. The fitting of effective mass of interlayer exciton, however, neglected uncertainties in ϕ as the sample to sample variation is the dominant contribution to fitting uncertainty.

To identify R- or H-stacking, we compare the second harmonic intensities from the monolayers with that from the bilayer. The second harmonic fields of the two layers will constructively interfere in heterobilayers with R-stacking (θ near 0° twist angle), but destructively interfere for H-stacking (60° twist angle), resulting in, respectively, stronger or weaker second harmonic signals in the bilayer compared to from monolayers [5]. Fig. 1 show the examples where the twist angles are measured to be 0.3° $\pm 0.2^\circ$ and 58.4° $\pm 0.2^\circ$ respectively.

Supplementary Note 4. Local inhomogeneity effect on SHG intensity and twist angle

Strain in the stacking process might result in the inhomogeneity of the SHG intensities and twist angles. To check the effect of strain, we have performed spatial mapping of the SHG over typical heterobilayers, by scanning the SHG pumping spot (of 1.5 μm in diameter) across the sample region. Typically quite uniform SHG signals across the sample in heterobilayer were measured when each monolayer was transferred without folding or breaking and with minimal visible bubbles. An example is shown in Fig. 2a and b. Based on the mapping, by only using heterobilayers with high structure integrity, we expect strain effect will not strongly affect the SHG signal within pumping spot of around 1.5 μm. Since SHG measurements require high-intensity lasers that may easily degrade the sample and it is time-consuming to perform a full spatial scan. For the heterobilayers in this work, we typically check two different positions on each bilayer and monolayer region to confirm consistency in determining the stacking order and twist angle. We have always obtained consistent results for the stacking order, and have found the twist angle varies within 0.2°, as shown by the example in Fig. 2c and d.

Supplementary Note 5. Fitting the reflection contrast spectrum

We calculate the reflection contrast spectrum using transfer matrix method. For the sample, we perform the transfer matrix calculation of white light reflection by a structure of four layers: hBN/WS_2 /$MoSe_2$ /sapphire substrate. For the substrate, we perform the calculation of two layers: hBN/sapphire, as shown by the schematic in Fig. 3a. And we get the reflection contrast spectrum $R_c = (R_{sample} - R_{sub})/R_{sub}$ from the transfer matrix calculation. The index of hBN is 2.1[6], and

sapphire is 1.47. The thickness of hBN is predicted by the contrast of microscope image calibrated by the AFM measurements. And we carefully fine tune the thickness of hBN within 5 nm to match the background of measured R_c away from the exciton resonance. We model the heterobilayer exciton resonances as Lorentzian oscillators:

$$\varepsilon = \varepsilon_B + \sum_{i=1}^{N} \frac{f_i}{E_i^2 - E^2 - iE\gamma_i}. \tag{7}$$

Here f_i and γ_i are the oscillator strength and the linewidth of the ith resonance [7]. ε_B is the background permittivity, taken to be 20[7, 8]. We mainly focus on the hybrid excitons in the energy region of $MoSe_2$ A exciton because they have narrower linewidths and are well isolated from other, higher energy resonances. To extract energies and oscillator strength of the hybrid states LHX_{MoA} and UHX_{MoA}, we perform least square fitting of the reflection contrast spectrum using transfer matrix calculations. To make the fit more accurate, we also include the resonances in the $MoSe_2$ B exciton region. So we fit the reflection spectra by least square method, and get the fitting errors with a 95% confidence interval. A typical example of the fit is shown in Fig. 3b. The fitted parameters are listed in the Table. S1 .

Supplementary Note 6. Noise level of reflectance contrast spectroscopy

The root mean square (RMS) noise of our RC spectra is estimated to be 1%. An example is shown by the RC spectrum of a WS_2 monolayer in Fig. 4. The RMS noise is calculated from a spectral range far from the A exciton of WS_2 , between 1.46 eV and 1.49 eV, as indicated by the red square and shown in the inset of the figure. To compare this noise level with measurable resonances, we shown in Fig. 4 a fit of the WS_2 A exciton. The inset shows three calculated RC spectra with an Lorentzian oscillator at the energy of 1.47 eV with linewidth of 10 meV, and with oscillator strengths reduced to 1/1000 (red), 1/100 (yellow), and 1/20 (purple) of the oscillator strength of WS_2 A exciton. While the A exciton resonance is very pronounced, any resonance with oscillator strength 2-3 orders of magnitude lower will be buried in noise, as shown by Fig. 4.

Supplementary Note 7. Photoluminescence from the hybrid excitons

We performed Photoluminescence (PL) measurement by pumping the sample using continuous wave diode laser at the energy of 2.33 eV. The laser power is 20 μW. As shown in Fig. 5, monolayer

$MoSe_2$ shows very strong trion emission and weak exciton emission because $MoSe_2$ crystal are typically n doped. From heterobilayer, we observed four peaks from PL spectrum, two of which agree with the resonance energies of the hybrid excitons identified by reflection spectrum. The other two peaks are around 30 meV red shifted from the two hybrid excitons, respectively, and disappear at higher temperatures, suggesting they are charged hybrid excitons. The upper and lower charged hybrid excitons are labeled as UHT and LHT respectively.

Supplementary Note 8. Heterobilayer with large twist angle misalignment

In bilayers twisted by more than 6°, the hybrid exciton doublets become hard to resolve in most of the samples, likely because the interlayer exciton is more blue detuned from the intralayer exciton and thus the UHX has a vanishing oscillator strength. Some examples are shown in Fig. 6.

Supplementary References

[1] Wu, F., Lovorn, T. & MacDonald, A. H. Topological exciton bands in moiré heterojunctions. *Phys. Rev. Lett.* **118**, 147401 (2017).

[2] Rasmussen, F. A. & Thygesen, K. S. Computational 2d Materials Database: Electronic Structure of Transition-Metal Dichalcogenides and Oxides. *J. Phys. Chem. C* **119**, 13169–13183 (2015).

[3] Wu, F., Lovorn, T. & MacDonald, A. H. Theory of optical absorption by interlayer excitons in transition metal dichalcogenide heterobilayers. *Phys. Rev. B* **97**, 035306 (2018).

[4] Zhang, L. *et al.* Highly valley-polarized singlet and triplet interlayer excitons in van der Waals heterostructure. *Physical Review B* **100**, 041402 (2019).

[5] Hsu, W.-T. *et al.* Second Harmonic Generation from Artificially Stacked Transition Metal Dichalcogenide Twisted Bilayers. *ACS Nano* **8**, 2951–2958 (2014).

[6] Stier, A. V., Wilson, N. P., Clark, G., Xu, X. & Crooker, S. A. Probing the Influence of Dielectric Environment on Excitons in Monolayer WSe $_2$: Insight from High Magnetic Fields. *Nano Letters* **16**, 7054–7060 (2016).

[7] Li, Y. *et al.* Measurement of the optical dielectric function of monolayer transition-metal dichalcogenides: MoS_2, $MoSe_2$, WS_2, and WSe_2. *Phys. Rev. B* **90**, 205422 (2014).

[8] Kim, K. *et al.* Band Alignment in WSe_2/Graphene Heterostructures. *ACS Nano* **9**, 4527–4532 (2015).

[9] Yu, H., Wang, Y., Tong, Q., Xu, X. & Yao, W. Anomalous light cones and valley optical selection rules of interlayer excitons in twisted heterobilayers. *Physical Review Letters* **115**, 187002 (2015).

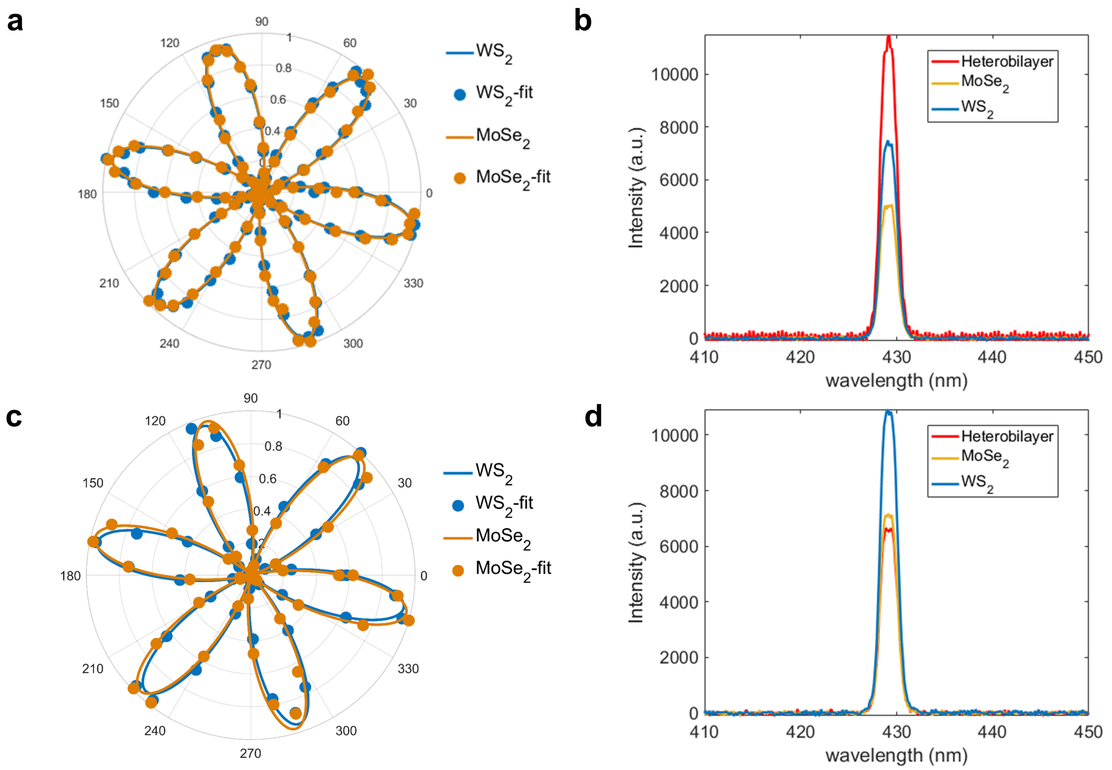

Supplementary Figure 1: Measurement of the hetero-bilayer twist angle. (a) The polarization-dependent second-harmonic-generation signal measured on the monolayer WS_2 (blue circles) and $MoSe_2$ (yellow circles) regions of the heterobilayer, and the corresponding fits with sinusoidal functions (blue and yellow lines). (b) The second harmonic signal from monolayer WS_2 , monolayer $MoSe_2$, and heterobilayer regions, measured with the same experimental configurations. (c)(d) Same measurements as described in (a) and (b), performed on a H-stacking sample.

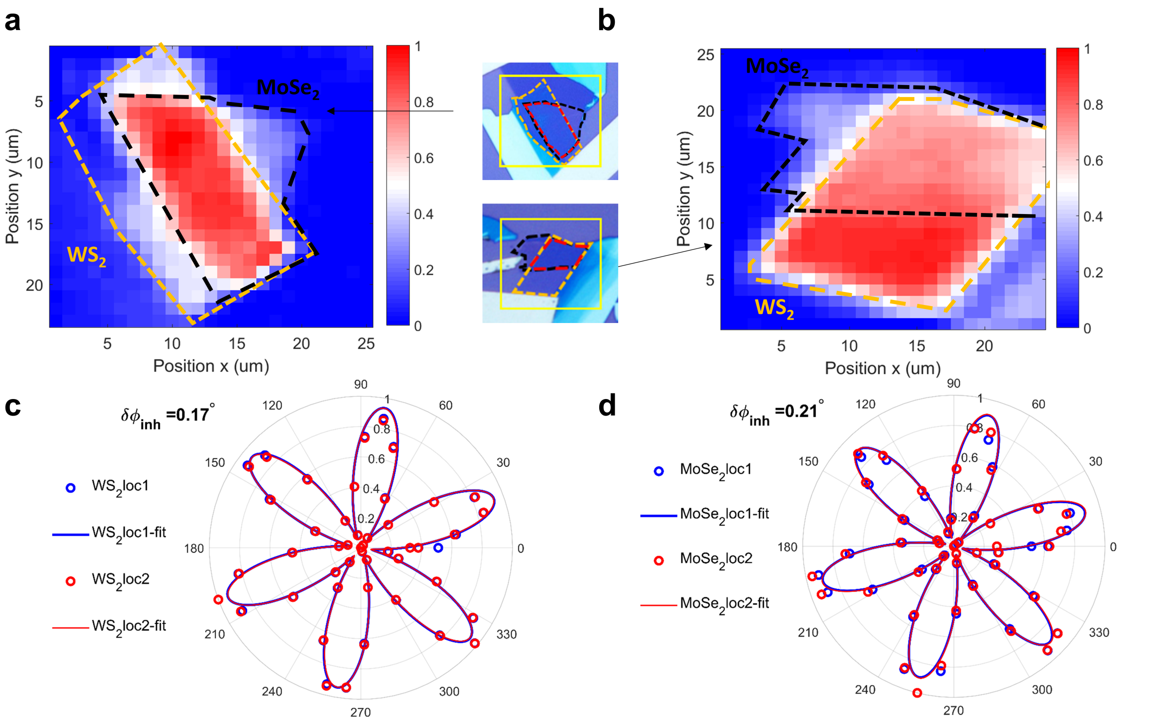

Supplementary Figure 2: (a) and (b) are spatial mapping of SHG over heterobilayers with R stacking order and H stacking order respectively. The yellow and black dashed lines label the two monolayers. The corresponding optical microscope images are in the middle. For the sample in a, the SHG shows stronger intensity at bilayer region as a result of constructive interference, indicating R stacking order. For the sample in b, the SHG shows weaker intensity at bilayer region as a result of destructive interference, indicating H stacking order. (c) and (d) show the variation of crystal orientation between two different locations measured on one monolayer WS_2 (c) and one monolayer $MoSe_2$ (d) respectively. Data taken from location 1 and location 2 are plotted in blue and red respectively.

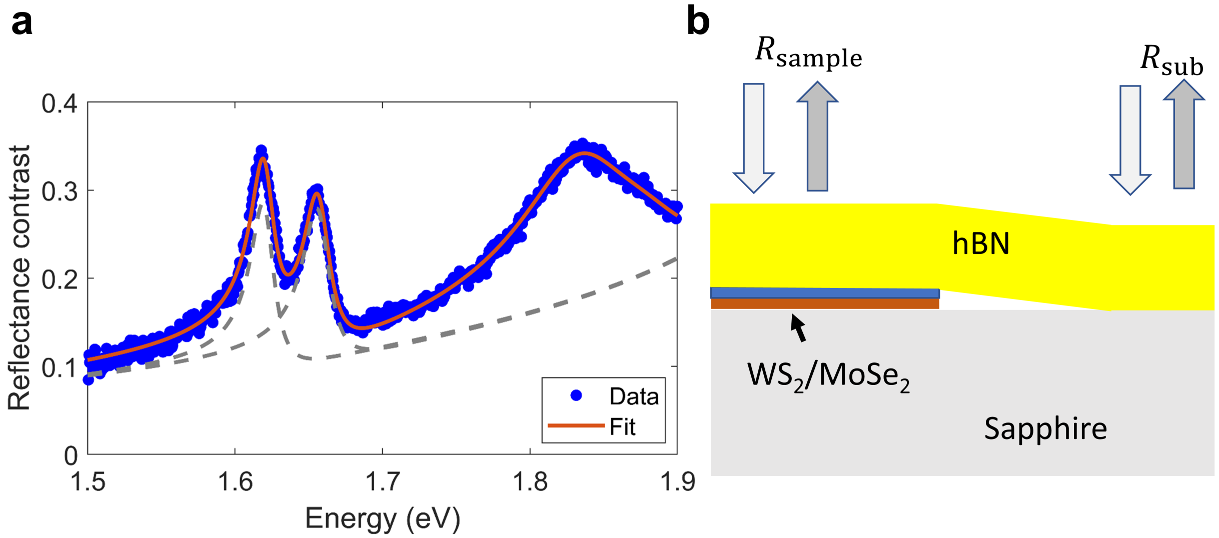

c

Hybrid Exciton	LHX_{MoA}	UHX_{MoA}	LHX_{MoB}	UHX_{MoB}
E (eV)	1.6208 ± 0.0002	1.6577 ± 0.0002	1.8389 ± 0.0055	1.8828 ± 0.0155
f (eV^2)	0.268 ± 0.006	0.278 ± 0.007	0.837 ± 0.439	0.516 ± 0.549
γ(eV)	0.0190 ± 0.0005	0.0214 ± 0.0006	0.08534 ± 0.0113	0.0960 ± 0.0458

Supplementary Figure 3: (a) Schematic showing how the reflection contrast spectrum is measured and simulated. See Note 5 of Supplementary Material for the details of the transfer matrix calculation.(b)An example of the RC spectrum fitted by transfer matrix calculation. Dots are data, solid line is the fit, and dashed lines are the fitted individual hybrid exciton resonances. (c)The parameters extracted by fitting the spectrum in (a).

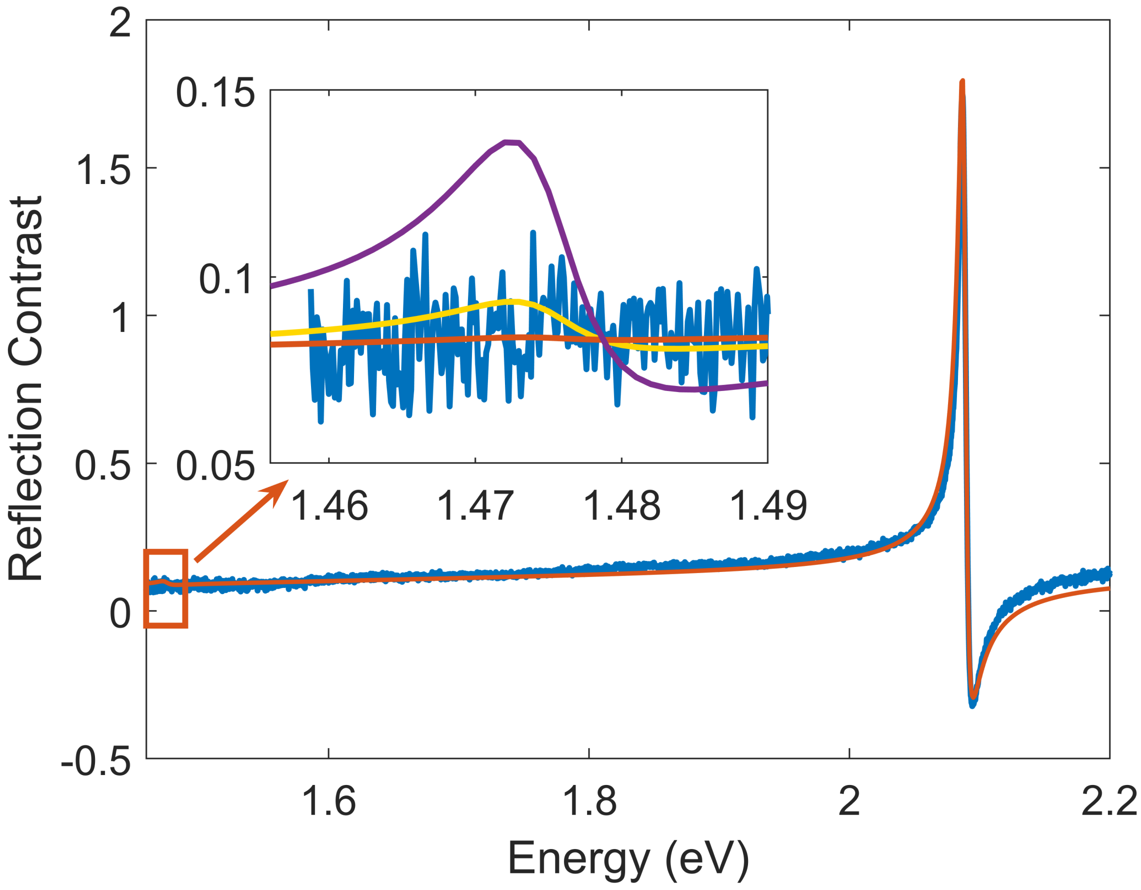

Supplementary Figure 4: The reflectance contrast (RC) spectrum of a WS_2 monolayer. Blue line is the measured spectrum, and red line is the fit. The inset shows a zoom-in of the spectral range marked by the rectangle, from which we estimate the noise background of the RC spectrum. The solid lines are the simulated RC spectra using a Lorentzian oscillator at the energy of 1.47 eV, with line width of 10 meV and with different oscillator strength: 1/1000, 1/100, and 1/20 of the WS_2 A excitons, which are plotted in Red , yellow, purple respectively.

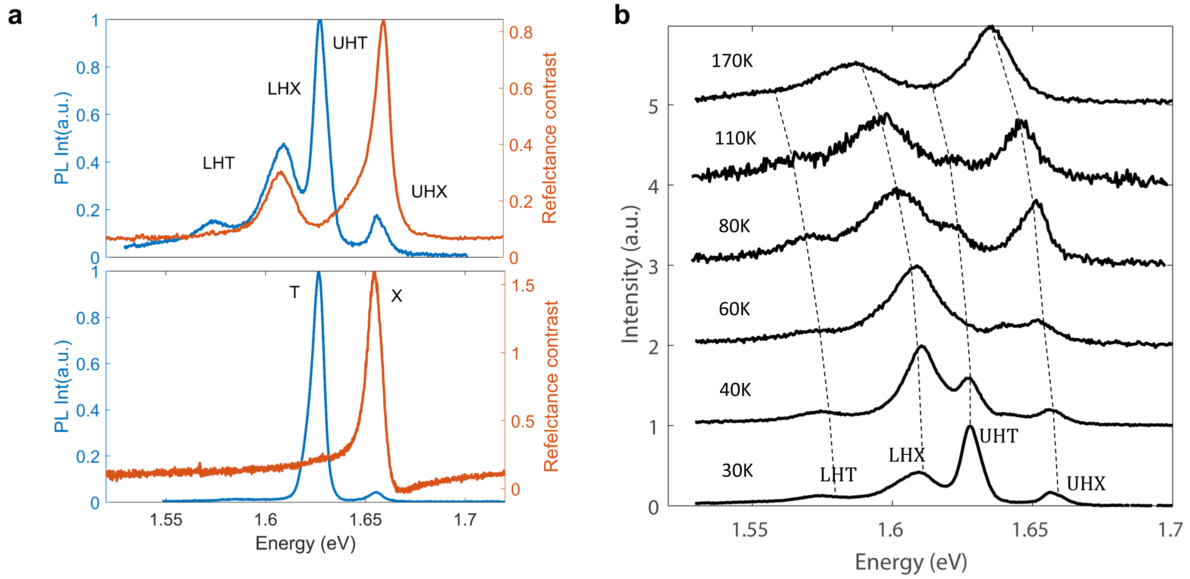

Supplementary Figure 5: PL from the heterobilayers. (a) PL (blue) and Reflectance contrast spectra (red) from a heterobilayer (top panel) and a MoSe2 monolayer (bottom panel). Upper and lower neutral hybrid excitons are labeled with UHX, and LHX respectively, and the charged hybrid exciton are labeled with UHT, and LHT. Exciton and trion from the $MoSe_2$ monolayer are labeled with X and T respectively. Trion can only be resolved in PL spectrum in both monolayer and heterobilayers due to much smaller oscillator strength. (b) Temperature dependence of the PL spectrum. With increasing temperature, all the four resonances red shift. UHT and LHT disappear at high temperature, as indicated by the dashed guidelines.

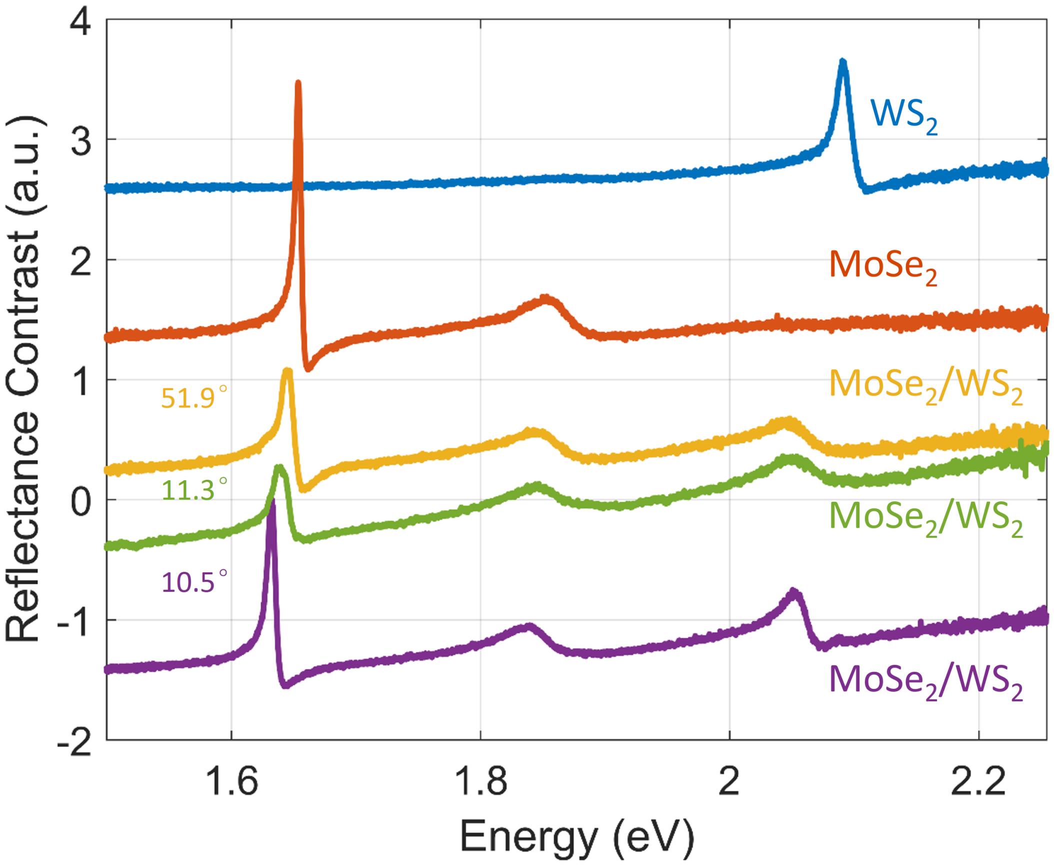

Supplementary Figure 6: RC spectra of heterobilayers with large twist angles (bottom three) compared with RC spectra from monolayer $MoSe_2$ and WS_2 (top two). Hybrid exciton doublets are not well resolved in these bilayers.
